# Dr. Jackson, on the Virtues of the Spider's Web

**Published:** 1809-05

**Authors:** Robert Jackson

**Affiliations:** London, No. 3, Panton-Square


### Medical and Physical Journal.

VOL. XXI.] MAY, 1809. [NO. 123.

*Printed for R. PHILLIPS, by W. Thorne, Red Lion Court, Fleet Street, London.*

### *To the Editors of the Medical and Physical Journal.*

GENTLEMEN,

AS you seem always ready to put before the public such hints or suggestions on medical subjects, as you may receive from persons of credibility and good intention, I am induced to submit to your consideration, a short notice of the effects of a remedy, which some may, perhaps, regard as too vulgar to be admitted in the physician's catalogue, or which others may be disposed to disparage, as it is not brought from a foreign country, or does not find a place in the apothecary's shop. The spider's web, (vulgarly cob-web) is known to many of the common people as a cure for ague and fever; but I believe that few of the regular practitioners have yet employed it on that, or any other account.

In the year 1800, the late Dr. Gillespie, physician in Edinburgh, a man of sound professional judgment, and of great candour and sincerity of character, mentioned to me, by accident, that once, when baffled by the obstinacy of an intermitting fever, he had recourse to this vulgar remedy; and with the most perfect success In the same year, and soon after I went to Chatham to the army depôt, I met with some intermittents, which, if suspended for a time by bark or arsenic, were still liable to recur, and which I did not on that account consider as effectually cured, or disposed to be effectually cured by these customary and ordinarily powerful means. But I shall state the case briefly, that you may be enabled to judge more correctly of the result. Serjeant Anderson, a respectable soldier, had been long harrassed by this complaint, and though he had been treated with all care and attention, there was yet no fair prospect from all the trials that had been made, that the disease was to be soon overcome. I was interested for the man on his own account, and I was also disappointed at the failure of my endeavours; for intermitting fever was a disease of which I had had much experience, and, in accomplishing the cure of which, I had seldom failed. The obstinacy of the case brought to my mind the fact which had been mentioned to me by Dr. Gillespie; and as I thought myself perfectly safe in giving credit to Dr. Gillespie's testimony concerning the power of the cobweb, I desired that some of that substance should be carefully collected from the cellars, and made into pills of five grains each. As I was desirous to ascertain the effect precisely, and from my own observation, I administered, with my own hand, one pill about two hours before I expected the recurrence of the paroxysm, and two more a few minutes before the usual time of the paroxysm's return. The patient was ignorant of the nature of the substance that was given to him, and thus could not be supposed to be acted upon through imagination; yet the effect was perfect. The usual periods past without return, or without the most remote feelings of it; and though I considered the disease as cured, yet as it had been of long continuance, and had considerably enfeebled the powers of the constitution by its continuance, I recommended a furlough on acconnt of change of air, so soon as I was satisfied that its course was completely arrested. Besides Anderson, there were then three others in the hospital ill of intermitting fevers. These cases were also untractable; and as the success of the cobweb had been so decided in the instance of Anderson, I was desirous to ascertain the extent of its virtues by farther experiment in theirs. I accordingly administered, with mine own hand, a pill to each of the persons alluded to, about two hours before I expected the return of the paroxysm, and two more a few minutes before the time at which it had been accustomed to return. The success was perfect in all the three.

On the faith of the instances which I have now mentioned, I was, and still am, disposed to consider cobweb as possessing the power of suspending the course of intermitting fever with great certainty; I shall not therefore trouble you with farther proof of the fact; but I must beg leave to state the history of a case, where it was given in the actual paroxysm with unexpected success; and I do this the more particularly, as the occurrences which I then observed, led me to reason on the subject, and to extend the the remedy to some conditions of disease to which I do not know that it had ever been before applied. A soldier of the Buffs, recently returned from the West Indies, was brought to the hospital, as having had a severe paroxysm of fever the preceding day. I have no note of what was done for him on the day on which he was received; but next day, while I was visiting the patients in the ward in which he lay, I observed the commencement of his paroxysm, and I perceived it was marked by such collapse and ghastliness, that, had I not known the nature of the case, I should have been apprehensive that the man was in the act of dying. I immediately gave him a cobweb pill, (for a dispenser of medicines attended while I visited, and cobweb pills were among the hospital *formulæ)*; in less than a minute he was perfectly tranquil; he even expressed comfort and satisfaction in himself that appeared unaccountable, and which I must confess surprised me, the change being sudden and complete, as if from the effect of a charm. The pulse was now calm and orderly; a very gentle but warm perspiration took place immediately, and health was instantly restored.

In reflecting on what happened under my own eye, in the case of the soldier of the Buffs, I could not help concluding, that cobweb possessed an extraordinary and altogether an unexplicable power in calming irritations, and in diminishing the excess of bodily torments; I therefore was induced to try it in other forms of febrile irritation, besides those belonging to intermittent, viz. in the deliria, pains, spasms, and subsultus common in fevers of the continued class. And having done so in various instances, I am warranted to say, that the effect far exceeded my expectations; it gave tranquillity, and sometimes perhaps saved life.

I have thus noticed, in a cursory manner, the power of cobweb in preventing the recurrence of the intermittent paroxysm, in allaying the irritations, and in suspending the course of the paroxysm when actually begun, and in tranquilizing patients who are irritable in the extreme in other conditions of fever, viz. outrageously delirious, or otherwise agitated in body and mind. I now farther add, that the trials were extended, and that perfect cures were effected in some troublesome spasmodic affections; even that respite was procured in others that were fatal in themselves, and that seemed to stand beyond the reach of other means. I have thus given cobweb, with the most marked benefit, in cases of dry, irritating coughs, usually termed nervous, sometimes singly, sometimes joined, and I think advantageously joined, with opium. I have also given it in the advanced stage of phthisis, where it procured a respite, of which I could scarcely have entertained expectation. I mention a case in detail, that you may obtain a better idea of the nature of the effect. A young man, of a consumptive family, was attacked with what was considered to be a cold, about last Easter. The complaint went on to increase, and when I saw him, which was in the month of August, phthisis was completely confirmed; the pulse was small and frequent, rarely, if ever, under one hundred strokes in a minute, sometimes far above that number; the flesh wasted fast; the strength was greatly impaired; the expectoration was in great quantity, and decidedly purulent; the cough was extremely troublesome; the voice hoarse, and withal, there were occasional pains, or stitches, in the sides and breast. The hectic was perfectly established, and no person could, in conscience, entertain any reasonable hopes of recovery. Emetics and blisters repeated at intervals, with other means commonly known and usually employed in similar cases, procured occasional respites; but they made no decided impression on the course and character of the disease. About the beginning of October, the distresses were urgent; the cough was almost incessant, so that the young man had scarcely any rest during the night; the pulse was so frequent, and at the same time so small, that it was scarcely possible to number it; the legs swelled as high as the knees, and the strength declined rapidly. His friends were anxious in the extreme for relief; and the apothecary who attended him, and who had exhausted all the means from which relief is usually obtained, wrote to me, soliciting strongly that I would think of something which might mitigate his sufferings, for to give a favourable turn to the course of the disease was not expected. The tranquilizing effects of cobweb occurred to me on this occasion; and I suggested to the apothecary, who was a man of discretion, to prepare some pills of five grains each, and to give one of them at bed-time, or at any other time when cough or pain was distressing. He did so, and in about ten days I received a letter from him, stating, that his patient was so much improved, that his friends were in great expectation of his doing well, and requested that I would visit him, (the distance was near thirty miles) to satisfy them on that head. I went to see him, in compliance with the request of the parents, and found, on inquiry, that he had rested tranquilly the first night after he took the cobweb, and tolerably well the most of the succeeding; that the violent irritation and distressing cough had abated; that the expectoration had not diminished materially in quantity, but that it came up easily; that the strength had improved, and that the swelling of the legs had nearly disappeared. The mitigation of the symptoms was obvious, but the disease still existed; I had no hopes that he could recover, but I was gratified to see that his pains and distresses were materially relieved.

The above history shews very clearly the extraordinary powers of cobweb in allaying irritations, and in procuring ease in a case past the reach of common remedies. To this I shall add a short notice of what I have observed in other cases of a somewhat different kind, and which also tend strongly to corroborate the extent of the tranquilizing effects of this substance. A gentleman of my acquaintance had for several months been extremely indisposed; the complaint complicated, but the nature of it not clearly understood. The joints, particularly the knees, were swelled, and extremely painful; the flesh was much wasted; there were frequent chills and occasional feverishness, especially towards evening; the pulse was small, irritated, and frequent; the appetite was, notwithstanding, better than might have been expected; the tongue was clean; the eye clear; the thirst considerable; there was evident hectic, but it was difficult to say on what it depended. There were suspicions, indeed marks of a venereal taint, and he was kept under the influence of mercury for six weeks or two months, during which time the pains in the joints increased; the flesh wasted fast; and he was reduced to a state of great weakness; distressed, at the same time, by restlessness and want of sleep. Fidgetting, irksomeness, and desire of change of posture were extremely annoying. In bed he had no rest, it not being in his power, during the greater part of the night, to keep his limbs in one posture for ten minutes at a time; nor was he sensible that he had slept two hours at once for the last three months. Having mentioned these circumstances to me one day when I called upon him as a friend, a recollection of what I had seen from the effect of cobweb presented itself to my mind, and I took an opportunity of suggesting to his physician, the propriety of making a trial of it, with a view to give the poor man a temporary respite from his sufferings. The physician accorded; and in prosecution of the suggestion, some pure cobweb was procured, and made

 into into pills of four grains each, and given by him, with directions to take one at bed-time, and another during the night, if he was not tranquil. He accordingly took one at ten, and, as he awoke at twelve, he took another, though he had felt no uneasiness. He rested calmly till morning; and, in repeating the pill afterwards, he uniformly found that, though he might not actually sleep, he was not only enabled but disposed to lie in one position till the morning. He observed to me that, when he took opium, his mind was engrossed with some foreign object, his attention was thereby diverted from his local pains and uneasinesses, but that he was much disordered next day; that when he took cobweb, of the nature of which he was ignorant, his mind was his own, but the sensations of pain and uneasiness were blunted, or obliterated, in a manner he did not understand, and that next day he felt no inconvenience of any kind. I mention this case, merely as an instance of the singular power which this substance possesses of diminishing excess of irritability, not as a remedy for the cure of a complicated organic disease.

The other instance which I shall trouble you with, occurred lately. In passing through one of our hospitals, in which there were many persons ill of infectious fever of various form, and in various stages of progress and degrees of intensity, the officer who had the charge of the establishment, asked my opinion concerning a person, for the safety of whose life he entertained serious apprehension. The disease was fever advanced in progress, and rather of the dysenteric form; the patient was much emaciated; the skin was dry, and not preternaturally hot; the pulse was small and frequent; the tongue dry, glossy, and unusually red; the stomach was rather distended, and, together with these symptoms, there was hiccough, troublesome and extremely distressing, as repressed by nothing that had been tried. I suggested that camphorated mixture, with a portion of white vitriol and nitre, should be given frequently, with the view of acting on the parched and inflamed surfaces of the mouth, gullet, &c. and that a pill of cobweb should also be given, with a view to arrest the hiccough. My recommendation was adopted, and the hiccough was restrained. It returned after some time, and was again restrained by the same means: the patient eventually recovered, though his situation had appeared to be extremely perilous.

I might multiply instances of the effects of this substance, but those I have now given will enable you to form some opinion concerning the value of its tranquilizing izing effects, and may perhaps induce some practitioners to make trial of it in hydrophobia, should an instance of that melancholy and hitherto fatal disease ever fall under their observation. The hydrophobia appears, by the best accounts which have been given of it, to be a disease of excessive irritability, and it is proved in the statements which I have given, the truth of the greater part of which I can yet attest by living evidence, that cobweb diminishes morbid irritability, and calms irritations of both body and mind, in a degree far exceeding any drug or remedy within the circle of our knowledge.

I am, &c.

ROBERT JACKSON, M. D.

*London, No.* 3, *Panton-Square, March* 20, 1809.

